# Anti-HBs Positivity Related to Past HBV Infection and Vaccination in Older Adults in Polish Population—Cohort-Based Study

**DOI:** 10.3390/vaccines13010018

**Published:** 2024-12-29

**Authors:** Katarzyna Zięba, Kacper Jagiełło, Joanna Musialik, Łukasz Wierucki, Adam Hajduk, Małgorzata Mossakowska, Jerzy Chudek

**Affiliations:** 1 Department of Internal Medicine and Oncological Chemotherapy, Medical University of Silesia in Katowice, 40-029 Katowice, Poland; chj@poczta.fm; 2 Department of Preventive Medicine and Education, Medical University of Gdańsk, 80-211 Gdańsk, Poland; kacper.jagiello@gumed.edu.pl (K.J.); wierucki@gumed.edu.pl (Ł.W.); 3 Department of Nephrology, Transplantology and Internal Medicine, Medical University of Silesia in Katowice, 40-055 40-027 Katowice, Poland; jmusialik@sum.edu.pl; 4 Department of Rheumatology, Clinical Immunology, Geriatrics and Internal Medicine, Medical University of Gdańsk, 80-214 Gdańsk, Poland; adam.hajduk@gumed.edu.pl; 5 Study on Ageing and Longevity, International Institute of Molecular and Cell Biology, 02-109 Warsaw, Poland; mmossakowska@iimcb.gov.pl

**Keywords:** hepatitis B, vaccination, HBV, older adults

## Abstract

Background: In Poland, a national hepatitis B (HBV) immunization program was introduced for neonates in 1996, and between 2000 and 2011, those born from 1986 to 1995 were vaccinated. Little is known about vaccination rates among adults born before 1986. This study aimed to determine the frequency of anti-HBs seropositivity rates related to vaccination and past HBV infection in older Poles. Methods: The HBV serological status was analyzed in 5781 (96.6%) of the PolSenior2 population-based cohort (60+) by assessing serum seropositivity for HBs antigen, anti-HBs, and anti-HBc antibodies. The survey was performed in 2018–2019 and included medical and socio-economic questionnaires, anthropometric measurements, and comprehensive geriatric assessment. Results: Serological status implying past hepatitis B and serological profile consistent with anti-HBV vaccination corresponded to 15.2% (95% CI: 13.4–17.0) and 25.2% (95% CI: 23.4–27.0) prevalences, respectively. Female gender, living in a town or city, having better education, and suffering from coronary artery disease, or depression independently increased the rate of past hepatitis B. On the other hand, being ‘white collar’ and self-reliant, having the ability to use the Internet, and past surgical procedures in the last 5-year period were factors associated with a higher vaccination rate. Conclusions: More than 15% of older adults in Poland present serological profiles suggesting past hepatitis B, and one-fourth anti-HBV vaccination. Being functionally independent, ‘white collar’, using the Internet, and having past surgical procedures are factors associated with a higher chance of being vaccinated. Nevertheless, a large group of older adults should be prophylactically vaccinated due to increased exposure to medical procedures.

## 1. Introduction

Chronic viral hepatitis caused by hepatotropic virus type B (HBV) is still an important cause of liver diseases in nonvaccinated adults. It is estimated that chronic HBV infection, demonstrated by the presence of hepatitis B surface antigen (HBsAg), affected 254 million people and caused 1,200,000 deaths in 2022 worldwide [1]. Fatality is especially caused by HBV-related liver cirrhosis and hepatocellular carcinoma (HCC) [2]. In developing countries, 60% of all liver cancer cases are caused by HBV infection. In developed countries, it accounts for 23% of cases [3].

The hepatitis B virus is transmitted by contact with infected blood or semen. Transmission routes include perinatal transmission from mother to newborn, sexual transmission, especially among people with multiple partners, or male–male sexual contact. In addition, dialysis or blood transfusions are still important sources, especially in developing countries. Other sources of infection include contaminated medical, surgical, or dental instruments [4]. The clinical course has two phases of infection: acute and chronic. For the acute phase, in addition to the presence of HBsAg, specific IgM antibodies against the hepatitis B virus core antigen (anti-HBc IgM) are found. In the chronic phase, the spectrum of HBV antigens and antibodies is detected, but HBsAg and anti-HBc IgG are always detected in serological tests. We can also diagnose chronic hepatitis B by detecting the presence of one of the above three markers in two tests with at least a six-month interval between them. If the above criteria for chronic or acute entities were not met, the case is classified as unspecified hepatitis B [5].

Prophylactic vaccination is a highly effective method of hepatitis B prevention. The level of anti-HBs was studied in Alaska residents 30 years after vaccination in 1981. Anti-HBs titer was determined, and the response to a booster dose was assessed if the anti-HBs level was <10 IU/L. A 30-year vaccination efficacy was estimated to be >90%, and no booster doses were needed [6].

In 1991, the World Advisory Group of the Expanded Program on Immunization (EPI) recommended the inclusion of hepatitis B vaccination in national immunization programs before 1995 in countries with an HBV prevalence of 8% or more and by 1997 in countries with a lower prevalence. At the end of 2014, newborn hepatitis B vaccination were introduced in 184 countries [7].

In Poland, mandatory vaccination of newborns and infants was introduced in the vaccination calendar in 1996, while the population born between 1986 and 1995 was vaccinated from 2000 to 2011 [8].

Data on the number of people vaccinated against hepatitis B in the Polish population born before 1986 are lacking. The vaccination of the non-vaccinated adult population is recommended before surgery but is not mandatory [9].

In Poland, the predominant HBV genotype is genotype A, found in 67% of people infected with the virus; the second most common genotype is genotype D, found in 20% of infected patients [10]. A huge number of adults were exposed to HBV, and viral hepatitis, mostly subclinical (non-jaundiced), even not being aware of it.

Higher vaccination rates are reported in groups exposed to HBV transmission, including healthcare professionals and hemodialysis patients. The percentage of healthcare workers vaccinated against hepatitis B reached 70.1% in Italy and 63.4% in the US [11,12]. Disproportionally, vaccination coverage of dialysis patients in the US in 2018, was only 33.2% and 15.3% in the age groups of 18–59 and ≥60 years, respectively [13].

According to the data from the World Health Organization, only 30.4 million people, which is 10.5% of all people living with hepatitis B, were aware of their infection, and 6.6 million were being treated in 2019 [1].

This study aimed to determine the frequency of anti-HBs-positivity related to past infection and vaccination in correlation to social and economic factors in an older adult population.

## 2. Material and Methods

The PolSenior2, a cross-sectional study, was implemented from 2017 to 2020, with a pilot in 2017 and surveys of respondents in subsequent years (2018–2019). The inclusion criterion was only age (over 60 years), while no exclusion criterion existed. The PolSenior2 study enrolled 5987 subjects aged 60 years and above, a representative sample of the community-dwelling Polish population. The sampling method was previously described [14]. In brief, it was based on a multistage stratified-cluster random strategy, performed in 78 strata. A three-stage sampling of the country’s population was used to select survey participants. The participants were randomly divided into equal groups in terms of age and gender. This procedure was intended to increase the statistical power of the analyses conducted in the oldest. The study protocol was approved by the Bioethical Committee of the Medical University of Gdansk and met the requirements of the Declaration of Helsinki. Individuals who provided informed and written consent to participate in the PolSenior2 study were included.

Research procedures were conducted by study nurses at the participants’ place of living over three visits, obtaining a 56% response rate. They included socio-economic questionnaires, geriatric tests and scales (e.g., the Geriatric Depression Scale, Instrumental Activities of Daily Living), anthropometric measurements, and blood sampling (the methodology of the PolSenior2 study was previously published in detail [14]).

The serological hepatitis B virus (HBV) status was determined in 5781 (96.6%) subjects by assessing the presence of HBs antigen (HBsAg), anti-HBs, and anti-HBc antibodies in the collected blood samples.

The medical survey contained questions concerning hepatitis B and vaccination against viral hepatitis: 1. Has your doctor ever diagnosed you with hepatitis B or C (so-called implantation jaundice)?; 2. Have you been vaccinated against hepatitis B? It has also included questions about the number of pregnancies and deliveries that could be potential risk factors for hepatitis B virus infection in women.

### 2.1. Laboratory Assessments

All analyses (HBsAg, anti-HBs, and anti-HBc) were performed in serum samples using Atellica Solution Immunoassay and Clinical Chemistry Analyzers and reagents from Siemens Healthineers (Forchheim, Germany) in Brus Laboratory in Gdynia—a central laboratory for the PolSenior2 study.

### 2.2. Comprehensive Geriatric Assessment and Selected Geriatric Scales and Tests

The initial examination included a Mini-Mental State Examination (MMSE), and only respondents who scored at least 19 points (without moderate-to-severe dementia) and were consequently able to understand the questions included in the 15-item Geriatric Depression Scale (GDS-15). Respondents obtaining 6–15 points were classified as having depressive symptoms.

Respondents’ functional status was assessed using Lowton’s scale for Instrumental Activities of Daily Living (IADL), as described previously [14]. The IADL scores of 8–18 points were classified as a dependency, 19–23 points as a partial dependency, and 24 points as independence [15]. Dependency and partial dependency were analyzed as one category.

Respondents were also evaluated for selected chronic diseases such as diabetes, hypertension, coronary artery disease (CAD), history of stroke, hospitalizations for heart failure, and cancer history based on reported data verified with medical documentation presented by the participant.

Individuals treated for diabetes and those with fasting serum glucose levels of at least 126 mg /dL were considered to have diabetes according to the Polish Diabetes Association guidelines from 2020 [16].

Arterial hypertension was diagnosed when, during the two visits, the mean systolic blood pressure was at least 140 mmHg and/or the mean diastolic blood pressure was at least 90 mmHg. If the subject had been taking antihypertensive drugs due to previously diagnosed hypertension during the last week preceding the blood pressure measurements, hypertension was diagnosed regardless of the blood pressure measurement results, in line with the European Society of Cardiology/European Society of Hypertension (ESC/ESH) guidelines from 2018 [17].

### 2.3. Data Analysis

**Figure 1:**
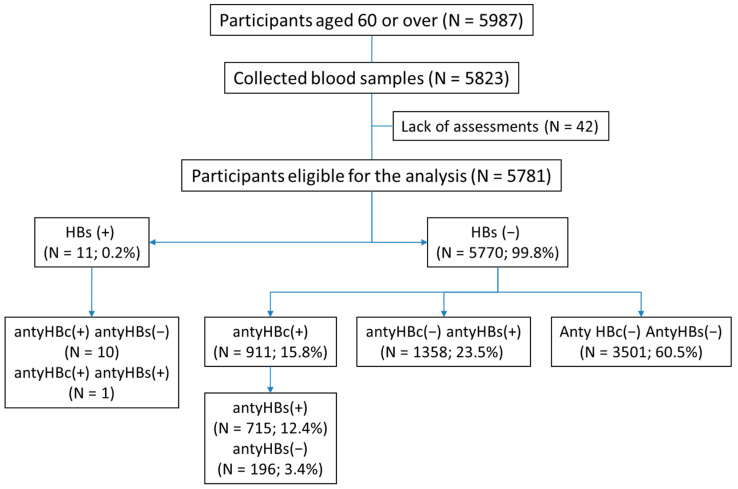
Study analysis flow chart.

Respondents were divided into three subgroups based on their HBV serological status: anti-HBc seropositive (anti-HBs seropositive or negative)—past hepatitis B infection; anti-HBs seropositive; and anti-HBc seronegative—past vaccination; and both anti-HBc and anti-HBs seronegative—unvaccinated and without past hepatitis B infection (Figure 1). The unvaccinated subgroup without past hepatitis B infection served as the comparator for the other subgroups. Fourteen HBsAg-positive subjects were excluded from further analysis as having active chronic hepatitis B.

The analysis included potential explanatory factors for being vaccinated and past hepatitis B: age, functional status, education, place of residence, region of residence, marital status and roommates, financial situation (enough money for food and clothes), and utilization of medical services during the last five years period, based on the data included in the socio-economic questionnaire.

### 2.4. Statistical Analysis

Counts and percentages were used to present information about categorical data. The continuous variable was presented using mean, standard deviation, median, and interquartile range. The post-stratification procedure (based on sampling weights) matched the age–sex sample distribution to the national population to estimate the prevalence of serological profiles with 95% confidence intervals. The between-subgroup differences were tested using the Mann–Whitney U test for quantitative data and the chi-square test for categorical variables. We applied logistic regression to assess the relationship between outcomes (the occurrence of anti-HBs antibodies related to vaccination and the co-occurrence of anti-HBs and anti-HBc antibodies after past hepatitis B) and a set of risk factors. Multivariable models were built based on a step-wise backward selection procedure and the Akaike information criterion. Regression coefficients were reported as odds ratios and 95% confidence intervals. The two-tailed tests were carried out with a significance level of *p* < 0.05. No alpha-level adjustment for multiple comparisons and testing was applied. Performing the statistical analysis, we assumed a random simple sample design instead of a complex sample design. Data visualization and statistical analysis were performed using the R statistical package (R Core Team, version 3.6.3.; Vienna, Austria; http://www.R-project.org/).

## 3. Results

### 3.1. Characteristics of the Subgroup with a Serological Profile of Past Hepatitis B

There were 911 study participants (15.8%) with serological status implying past hepatitis B. Based on this, we estimated the prevalence of anti-HBc seropositivity in Polish older adults to be at 15.2% (95% CI: 13.4–17.0).

The subgroup of anti-HBc contained significantly more women, better educated, ‘white collar’, and residents of large cities, as shown in Table 1. Individuals who were anti-HBc seropositive were more likely to live with another household member. Based on the Geriatric Depression Scale (GDS), depression was diagnosed in 33.4% of the subjects and was more frequent than in the anti-HBc and anti-HBs seronegative subgroup (27.5%). Of note, 42.2% of the respondents in the anti-HBc seropositivity subgroup claimed to be vaccinated against hepatitis B.

Age, marital and functional status, personal financial resources, ability to use the Internet, and utilization of medical services (frequency of visits to the general practitioner, hospitalizations during the last 5-year period) were similar in anti-HBc and anti-HBs seronegative subgroups. However, respondents in the anti-HBc seronegative subgroup had a higher rate of depression and coronary artery disease than the anti-HBc and anti-HBs seronegative subgroup.

In univariate logistic analysis (Table 2), female sex, city dwelling, solitary habitation, better education, ‘white collar’, coronary artery disease, and depression were associated with serological status implying past hepatitis B. In the multivariate logistic analysis, female gender, living in a town or city, better education, coronary artery disease, and depression independently increased the chance of past hepatitis B (Table 2).

### 3.2. Characteristics of the Subgroup with a Serological Status Characteristic for Vaccinated Against Hepatitis B

The serological profile of anti-HBV vaccination was noted in 1358 participants (23.5%, Figure 1), corresponding to 25.2% (95% CI: 23.4–27.0) prevalence in Polish older adults. This subgroup was significantly younger (by 2 years) and included more women, better educated, ‘white collar’, and residents of large cities than both other subgroups. Individuals who were anti-HBs seropositive were most likely to use the Internet and reported having sufficient personal incomes. Of these individuals, 69.3% of participants reported anti-HBV vaccination. This subgroup utilized consultations with specialists significantly more often and reported the highest rate of hospitalizations or surgery in the last 5-year period. There was also a slightly higher frequency of visits to general practitioners than in the anti-HBc and anti-HBs seronegative subgroup. Individuals in this subgroup presented the highest rate of independence in daily living and lower rates of diabetes and past stroke. The marital and living statuses were irrelevant.

In univariate logistic analysis (Table 3), female gender, better education, ‘white collar’, ability to use the Internet, utilization of medical services including visits to specialists, hospitalizations, and surgical procedures in the last 5-year period, and independence in IADL (Instrumental Activities of Daily Living) were factors associated with being vaccinated against hepatitis B. Factors that decreased the chance of being vaccinated were age over 75, living in a village, having diabetes and history of stroke. In multivariate analysis, ‘white collar’, ability to use the Internet, past surgical procedures in the last 5 years, and self-reliance were independent factors associated with a greater chance of vaccination. Factors that decreased the chance of being vaccinated were diabetes and a history of stroke (Table 3).

## 4. Discussion

Data obtained in the PolSenior2 study expanded our knowledge regarding anti-HBc-positivity related to past hepatitis B, estimated at 15.2% (95% CI: 13.4–17.0) in older Poles, and identified the following risk factors: female gender, city dwelling, better education, coronary artery disease, and depression. In addition, we estimated the anti-HBs positivity related to vaccination to be at 25.2% (95% CI: 23.4–27.0) of the older adult population in Poland and showed the following factors that favor the vaccination: independency in IADL, being ‘white collar’, with the ability to use the Internet, and a history of past surgical procedures during the last 5 years. Of those, only 69.3% reported anti-HBV vaccination, which may be caused by the frequent occurrence of memory disorders in this age group.

The high proportion of anti-HBc positive as the result of past HBV infection (15.2%) may be related to various routes of viral transmission existing in the past decades, including mother-to-child transmission, blood transfusions, medical procedures, and dental services, performed much longer than during the last 5-year period included in the study questionnaire. Less probable, in this old cohort, was the use of stimulants, such as intravenous drugs by adolescents and young adults, that became available in the eighties of the last century.

It should be noted that in the past, the rate of acute infections caused by HBV transmission related to hospital procedures reached up to 67% [18]. Numerous procedures and improvements were made to reduce the transmission of the virus in dental offices and hospitals, including the use of disposable needles, single-use medication containers, procedures for sterilizing instruments, and special attention to patient and medical staff hygiene [19]. Special attention was drawn to certain groups of patients (e.g., past exposure to infection) based on their past medical history. The assessment of anti-HBs status became routine before invasive procedures (also endoscopic) to avoid legal claims. The risk of infection transmission by using disposable accessories during, e.g., endoscopic procedures, was further reduced [20,21].

The risk of transmission of HBV through blood transfusions is currently extremely low (the estimated risk is less than 1:1,000,000) [22]. In 2024, experts reported on the extent of viral infections attributable to treatment with blood and blood products in the UK between 1970 and 1998. It stated that it was not possible to estimate the number of people infected with HBV due to limited data. It noted that hepatitis B testing for donors and plasma fractionations was introduced between 1971 and 1972. However, the tests at that time were still imperfect, precluding detection of all infected individuals [23].

Currently, all EU/EEA countries have control systems in places where donations are tested, using at least serological methods for HBV and HCV infections. The target for 2025 of 100% of blood units tested for bloodborne diseases is currently being met in 19 of the 20 countries that provided data [24].

Global data on the association between the prevalence of a history of past hepatitis B and coronary heart disease are inconsistent. It is reported that there is no association or a reduction in the proportion of individuals with past hepatitis B and CAD. In our study, CAD was an independent risk factor for HBV history. This may be a consequence of a long history of coronary interventions in some seniors. It was shown that the older population has a higher risk of undergoing invasive procedures, which could explain the occurrence of past infections related to these procedures, including the transmissions provider-to-patient in the pre-vaccination era [25,26,27].

It was proven that patients with chronic hepatitis B are more frequently depressed and their quality of life deteriorates. However, to our knowledge, this aspect has not been studied in patients recovering from hepatitis [28,29].

In this study, the prevalence of depression in the older population with a history of past hepatitis B was higher than in the group without a history of hepatitis B and who were not vaccinated. This interesting observation necessitates verification in other cohorts.

There is no better protection against HBV infection than vaccination. However, as shown by our study the vaccination rate against HBV in older Poles remains low (25.2%)—highlighting the further need for active campaigns to promote vaccination. European data concerning percentages of vaccinated against hepatitis B in the older populations are missing. Therefore, the low rate of vaccinated older adults may be an unnoticed problem in healthcare systems in other countries.

Factors favoring vaccination in old and very old Poles were mostly related to greater awareness of the potential risk of infection, associated with invasive medical services in better-educated ‘white collar’ and those using the Internet services. Consequently, the recommended vaccination before surgical procedures was strongly associated with being vaccinated. Better education is related to using the Internet and other digital devices and wider access to information. This results in increased awareness among this group and a higher vaccination rate. In addition, independence in IADL was found an important association with vaccination status. This may be related to higher utilization rates of medical services by more fit people.

Having diabetes and a history of stroke were independent factors reducing the chance of being vaccinated. This may have been associated with less care for health among diabetics, while past stroke may cause disability restricting access to preventive health services. So, this group of people might become of interest to GPs for preventive vaccination due to the need for increased frequency of medical services in these patients.

Currently, acute hepatitis B is a rare disease, while chronic infections are still being diagnosed in Poland. In 2021, 1547 cases of hepatitis B were registered, including 10 cases of acute hepatitis B (2 of them were imported). The detection rate of chronic and indeterminate cases was 4.03 per 100,000 population. The highest rate of new diagnoses of chronic or unknown-phase cases was 9.48/100,000 in the 30–34 age group, and higher among women. Among men, the highest detection rate (9.37/100,000) was in the 50–54 age group. The effects of the COVID-19 pandemic related to the detection of new cases (the rate was lower during the pandemic) are currently being offset. Due to the high migration of Ukrainian citizens to Poland, the epidemiological situation may have changed in recent years [30].

At this point, it is important to clarify the limitations of the performed analysis, concerning used definitions. Two main aspects should be noted. The first is the formation of anti-HBs antibodies. These can be formed either by the mechanism of inoculation (passive immune response) or past infection (cessation of primary infection and acquisition of immunity to reinfection). Currently, immune individuals are considered to be immune when the presence of anti-HBs antibodies in serum occurs in titers > 10 IU/L (concentrations of 1–100 IU/L) determine a low level of protection, and titers > 100 IU/L represent a high protection level. Still, it is considered that a high protection level is achieved after three doses of the vaccine [31]. However, a vaccinated person may lose anti-HBs antibodies over the years/decades [32]. Factors that interfere with the immune response include old age, kidney failure, or metabolic diseases such as diabetes [33,34,35]. Thus, the PolSenior2 study population has numerous risk factors that can affect the presence of antibodies and the degree of immunity to HBV infection. It is worth noting that we do not have data on the number and concentrations of doses administered, the type of vaccine used, and less importantly routes of administration. These data were not collected in the study questionnaire. In addition, the percentage of individuals non-responsive to vaccination remains unspecified.

Some individuals present anti-HBs antibody titer below 10 IU/L 4–8 weeks after vaccination. Among these individuals, according to in vitro studies, the ability to produce antibodies is lower, and immune immunity is deficient [35]. Furthermore, the lack of detectable titer of anti-HBs antibodies does not exclude existing immunity, and the ability to rapidly multiply antibodies after contact with the virus [36]. At the same time, some people with detectable antibodies present no immunity to HBV, as the antibodies are incapable of neutralizing the virus (unable to recognize the tertiary structure of the “a” determinant of the mutant HBs antigen or in other regions of the pre-s/s gene) [37]. Last but not least, we cannot exclude the possibility of false detections of anti-HBs antibodies in the bloodstream due to non-specific reactivity with interfering substances, which may include infectious agents or antibodies present after multiple transfusions [38].

Despite these study limitations, the percentage of anti-HBs antibodies present in the absence of anti-HBc antibodies and HBs antigen remains low (23.5% of the study population).

Another aspect arising from the study is the possible existence of occult hepatitis B infection (OBI). This is a condition in which replication-competent HBV DNA is present in hepatocyte cells, with or without HBV DNA in the blood, in individuals without HBsAg. We can divide OBI into seropositive and seronegative, depending on whether or not antibodies (anti-HBc and anti-HBs) are present [39]. Since viral load determinations are not routinely performed, those with negative HBs antigen and positive anti-HBc antibodies (15.8% in this study) should be considered as a target group for expanded diagnosis of latent HBV infection. The early detection of OBI is important due to the possibility of infection reactivation during immunosuppressive therapies or oncological treatments.

The detection of HBs antigen and anti-HBs antibodies in the older population could also result from the increasing number of hospitalizations and injuries at this age, and thus exposure to potential infection. This would tie in with the WHO’s Global Health Sector Strategies for HIV, Viral Hepatitis, and Sexually Transmitted Infections 2022–2030 by finding infected people, implementing treatment for them, and vaccinating those at risk of infection [40].

At this point, we emphasize the need to strive for HBV elimination from the environment. Among the measures that could be fundamental in this particular population is active screening by general practitioners, who have the most frequent contact with seniors reporting a lack of vaccination in the past. Such screening will allow for the vaccination of seronegative individuals and possibly identify infected, revealing the virus reservoir. These measures will reduce the long-term financial burden related to therapy for chronic complications. Our study showed that the actions taken to eliminate the virus must include the senior population.

The performed analysis has some limitations. There are no data on blood transfusions, cardiological services, past hospitalizations, or surgical procedures performed over a period longer than the five years included in the analysis. Moreover, the types of surgeries performed during the last 5-year period were not collected**.** However, currently, the use of medical services, including blood transfusions**,** is not a risk factor for HBV transmission and appears safe for patients. Information on vaccination came from the participant’s reports and was not verified with medical records. We would also like to point out that depression and cognitive impairment were diagnosed based on screening tests only. These, however, allowed for the separation of a group with severe cognitive impairment, who cannot be screened for depression with the GDS-15 scale. We acknowledge that diagnosing depressive disorders is challenging, as the presence of depressive symptoms is not always the same as the diagnosis of the disease and does not replace psychiatric evaluation. The usefulness of the Polish version GDS-15 scale in diagnosing depressive disorders has not been validated; however, it is used in daily clinical practice and repeatedly in numerous studies, including the first PolSenior study [41], and is widely used in geriatric populations worldwide [42,43,44].

## 5. Conclusions

More than 15% of older adults in Poland present serological profiles of antibodies suggesting past hepatitis B, and one-fourth corresponds to anti-HBV vaccination. Being functionally independent, ‘white collar’, using the Internet, and having past surgical procedures are factors associated with a higher chance of being prophylactically vaccinated. Due to the low vaccination rate and high rates of serological profiles of past infections in older adults, health services should support general practitioners in HBV testing. This will allow for identifying candidates for prophylactical vaccination and those at risk of infection reactivation during immunosuppressive and oncological therapies.

## Figures and Tables

**Table 1:** Comparison of subjects with defined serologic status for hepatitis B. Subgroups: anti-HBc (+)—past hepatitis B infection [A]; anti-HBc (−) and anti-HBs (+)—the past vaccination [B]; and anti-HBc (−) and anti-HBs (−)—unvaccinated and without past hepatitis B infection [C]. Data presented as numbers (%) or median (Q1–Q3).

	Anti-HBc (+) [A] *N* = 911	Anti-HBc (−) Anti-HBs (+) [B] *N* = 1358	Anti-HBc (−) Anti-HBs (−) [C] *N* = 3501	*p* A vs. C	*p* B vs. C
Age [years] *	74 (68–82)	72 (66–80)	74 (67–82)	0.55	<0.001
>75 years [N (%)]	422 (46.3)	516 (38.0)	1629 (46.5)	0.91	<0.001
Sex [N (%)]					
Women	508 (55.8)	801 (59.0)	1625 (46.4)	<0.001	<0.001
Men	403 (44.2)	557 (41.0)	1876 (53.6)		
Place of residence [N (%)]					
Village	279 (30.6)	429 (31.6)	1337 (38.2)	<0.001	<0.001
Town < 50,000 citizens	246 (27.0)	328 (24.2)	889 (25.4)		
City > 50,000 citizens	386 (42.4)	601 (44.2)	1275 (36.4)		
Marital status [N (%)]					
Never married/divorced/separated/ widowed	382 (42.3)	510 (38.0)	1345 (38.9)	0.06	0.55
Married	521 (57.7)	833 (62.0)	2111 (61.1)		
Educational level [N (%)]					
Vocational, primary or lower	461 (50.9)	612 (45.3%)	2000 (57.3)	<0.001	<0.001
Secondary or higher	445 (49.1)	740 (54.7)	1489 (42.7)		
Type of work (current or in the past) [N (%)]					
‘White collar’	281 (34.2)	504 (40.2)	912 (28.4)	<0.01	<0.001
‘Blue collar’, farmer or other	541 (65.8)	751 (59.8)	2294 (71.6)		
Using the Internet [N (%)]	306 (33.7)	570 (42.2)	1074 (30.9)	0.11	<0.001
Utilization of medical services during the last 5-year period [N (%)]					
At least 1 visit per year to GP	847 (97.1)	1279 (98.2)	3220 (96.8)	0.60	<0.05
Visits to specialists	639 (70.5)	1057 (78.4)	2459 (70.8)	0.85	<0.001
Hospitalization	417 (45.9)	677 (50.1)	1519 (43.6)	0.22	<0.001
Surgical procedures	281 (31.9)	502 (38.3)	990 (29.4)	0.15	<0.001
Diabetes mellitus [N (%)]	251 (27.6)	300 (22.1)	951 (27.2)	0.83	<0.001
Hypertension [N (%)]	699 (77.0)	1026 (75.6)	2716 (77.7)	0.66	0.13
Coronary artery disease [N (%)]	113 (12.7)	128 (9.7)	320 (9.4)	0.01	0.38
Past stroke [N (%)]	79 (8.7)	90 (6.6)	354 (10.1)	0.26	<0.001
Heart failure [N (%)]	206 (22.9)	253 (18.9)	739 (21.4)	0.32	0.06
Cancer survivors [N (%)]	59 (6.5)	100 (7.4)	219 (6.3)	0.98	0.22
Independent in IADL—24 pts [N (%)]	575 (63.1)	988 (72.9)	2212 (63.3)	0.90	<0.001
Depression in GDS—6–15 pts [N (%)]	304 (33.4)	347 (25.6)	963 (27.5)	<0.001	0.17
Reported anti-HBV vaccination [N (%)]	384 (42.2)	941 (69.3)	1145 (32.8)	<0.001	<0.001

* Median values with interquartile range, GP—general practitioner, IADL—Instrumental Activities of Daily Living (Lawton and Brody, 1969 [15]), GDS—Geriatric Depression Scale.

**Table 2:** Factors associated with the occurrence of anti-HBc seropositivity (past hepatitis B) [subgroup A] compared to the unvaccinated without past hepatitis B infection [subgroup C]. Univariate and multivariate logistic regression analyses.

	Univariate		Multivariate	
	OR (95% CI)	*p*	OR (95% CI)	*p*
Age > 75 years	0.99 (0.86–1.15)	0.91	-	
Sex				
Men	Ref		Ref	
Women	1.46 (1.26–1.69)	<0.001	1.45 (1.24–1.68)	<0.001
Place of residence				
Village	Ref		Ref	
Town < 50,000 citizens	0.91 (0.76–1.10)	0.33	1.25 (1.02–1.53)	0.03
City > 50,000 citizens	1.45 (1.22–1.72)	<0.001	1.37 (1.14–1.65)	0.001
Marital status				
Married	Ref		Ref	
Never married/divorced/separated/widowed	1.15 (0.99–1.34)	0.06	-	
Living alone	1.29 (1.08–1.54)	0.005	-	
Educational level				
Vocational, primary, or lower	Ref		Ref	
Secondary or higher	1.30 (1.12–1.50)	<0.001	1.21 (1.03–1.42)	0.02
Type of work (current or in the past)				
‘Blue collar’, farmer, or other	Ref		Ref	
‘White collar’	1.31 (1.11–1.54)	0.001	-	
Using the Internet	1.14 (0.97–1.33)	0.10	-	
Utilization of medical services during the last 5-year period				
At least 1 visit per year to GP	1.12 (0.72- 1.75)	0.60	-	
Visits to specialists	0.98 (0.84–1.16)	0.84	-	
Hospitalization	1.10 (0.95–1.27)	0.22	-	
Surgical procedures	1.13 (0.96–1.32)	0.15	-	
Diabetes mellitus	1.02 (0.87–1.20)	0.82	-	
Hypertension	0.96 (0.81–1.14)	0.65	-	
Coronary artery disease	1.40 (1.11–1.76)	0.004	1.42 (1.13–1.79)	0.003
Past stroke	0.85 (0.66–1.09)	0.20	-	
Heart failure	1.09 (0.92–1.30)	0.31	-	
Cancer survivors	1.03 (0.77–1.39)	0.83	-	
Independent in IADL (24 pts)	0.99 (0.85–1.14)	0.90	-	
Depression in GDS (6–15 pts)	1.32 (1.13–1.54)	<0.001	1.35 (1.14–1.59)	<0.001

Ref—reference, GP—general practitioner, IADL—Instrumental Activities of Daily Living (Lawton and Brody, 1969 [15]), GDS—Geriatric Depression Scale.

**Table 3:** Factors associated with the occurrence of anti-HBs seropositivity among anti-HBc negative (past vaccination) [Subgroup B] compared to the unvaccinated and without past hepatitis B infection [Subgroup C]. Univariate and multivariate logistic regression analyses.

	Univariate		Multivariate	
	OR (95% CI)	*p*	OR (95% CI)	*p*
Age > 75 years	0.70 (0.62–0.80)	<0.001	-	
Sex				
Men	Ref		Ref	
Women	1.66 (1.46–1.88)	<0.001	-	
Place of residence				
City > 50,000 citizens	Ref		Ref	
Town < 50,000 citizens	0.78 (0.67–0.92)	0.003	-	
Village	0.68 (0.59–0.79)	<0.001	-	
Marital status				
Married	Ref		Ref	
Never married/divorced/separated/widowed	0.96 (0.84–1.09)	0.55	-	
Living alone	1.14 (0.98–1.33)	0.09	1.11 (0.94–1.32)	0.22
Educational level				
Vocational, primary, or lower	Ref		Ref	
Secondary or higher	1.62 (1.43–1.84)	<0.001	-	
Type of work (current or in the past)				
‘Blue collar’, farmer or other	Ref		Ref	
‘White collar’	1.69 (1.47–1.93)	<0.001	1.46 (1.26–1.70)	<0.001
Using the Internet	1.64 (1.44–1.86)	<0.001	1.24 (1.06–1.46)	0.007
Utilization of medical services during the last 5-year period				
At least 1 visit per year to GP	1.57 (1.14–2.77)	0.01	-	
Visits to specialists	1.50 (1.29–1.74)	<0.001	-	
Hospitalization	1.30 (1.14–1.47)	<0.001	-	
Surgical procedures	1.49 (1.31–1.71)	<0.001	1.50 (1.30–1.74)	<0.001
Diabetes mellitus	0.76 (0.66–0.88)	<0.001	0.82 (0.62–0.97)	0.02
Hypertension	0.89 (0.77–1.03)	0.12	-	
Coronary artery disease	1.03 (0.83–1.28)	0.77	-	
Past stroke	0.63 (0.49–0.80)	<0.001	0.69 (0.53–0.91)	0.008
Heart failure	0.86 (0.73–1.01)	0.06	0.99 (0.83–1.19)	0.93
Independent in IADL (24 pts)	1.56 (1.35–1.79)	<0.001	1.28 (1.08–1.52)	<0.001
Depression in GDS (6–15 pts)	0.90 (0.78–1.04)	0.17	-	

Ref—reference, GP—general practitioner, IADL—Instrumental Activities of Daily Living (Lawton and Brody, 1969 [15]), GDS—Geriatric Depression Scale.

## Data Availability

The data supporting the findings are available from Łukasz Wierucki upon reasonable request.
